# Dual polarity open circuit voltage in triboelectric nanogenerators originated from two states series impedance

**DOI:** 10.1186/s11671-024-04056-y

**Published:** 2024-07-06

**Authors:** Jiwon Jeong, Jiyoung Ko, Jongjin Lee

**Affiliations:** Department of Physics and Research Institute of Natural Science, Gyeongsang National University, Jinju, 52828 South Korea

**Keywords:** Triboelectric nanogenerator, Open-circuit voltage, Asymmetric voltage, Variable impedance energy device, Measurement system

## Abstract

Experimental and simulation studies demonstrated that the initial voltage setting significantly influences the open-circuit voltage (*V*_*OC*_) in triboelectric nanogenerators (TENGs). Utilizing diode configurations, we consistently observed two distinct *V*_*OC*_s independent of the initial settings. A lower *V*_*OC*_ corresponded to the surface voltage (*V*_*Surface*_), while a higher *V*_*OC*_ was amplified by the product of the *V*_*Surface*_ and the TENG's characteristic impedance ratio. Notably, a lower measurement system capacitance provided a more precise representation of the inherent characteristics of the TENG. Conversely, an increase in system impedance led to a convergence of the two *V*_*OC*_s and a reduction in their magnitudes relative to *V*_*Surface*_. These findings suggest that optimizing the initial/repeated charge balancing and minimizing capacitive loads are crucial for maximizing TENG output power in practical applications.

**Supplementary Information:**

The online version contains supplementary material available at 10.1186/s11671-024-04056-y.

## Introduction

As low-power technology advances, electronic components are becoming indispensable across a broad spectrum of industries. Energy harvesting is an essential field of research for obtaining sustainable energy sources for low-power electronic components [1–3]. Among the various energy harvesting technologies, a triboelectric nanogenerator (TENG) directly converts input energy into electrical energy through triboelectric and electrostatic induction [4–6]. As an effect of direct conversion, when converting small amounts of ambient energy into electricity, TENGs are more efficient than conventional electromagnetic induction generators [7, 8]. Furthermore, miniaturization and efficient maintenance can provide flexibility in various applications, such as providing a sustainable power source for active sensors [9, 10], biomedical applications [11, 12], and building off-grid networks in hard to manage locations [13, 14].

The output of electrical energy devices can be quantified by two key parameters: current, which is the amount of moving charge, and voltage, which is the difference in the potential energy of these moving charges. For this reason, the output performance of an electrical energy device is evaluated in terms of the open-circuit voltage (*V*_*OC*_), which refers to the maximum voltage a device can produce, and the short-circuit current (*I*_*SC*_), which refers to the maximum current that a device can support. Conventional energy devices have a fixed intrinsic output impedance, which renders a single *V*_*OC*_ and *I*_*SC*_. In the case of TENGs, the device has a variable output impedance depending on the motion, resulting in an electrostatic displacement current between the facing two conducting plates. The TENG output generated by impedance change can exhibit asymmetrical characteristics [15–17]. In order to understand the output characteristics of the TENG, it is necessary to consider the changing output impedance. Many studies have reported enhancing the output of TENGs by optimizing the output impedance [18–20].

In this study, we analyzed the two distinct *V*_*OC*_s generated at the two extreme output impedances of the maximum and minimum states. Experimental and circuit simulations confirmed the existence of these two *V*_*OC*_s. Circuit simulation revealed that these two *V*_*OC*_s were measured differently depending on the input capacitance of the high-impedance measurement system. This paper presents an analysis of the methods and principles of operation for exploiting the variable output impedance effects of TENGs. Our findings will provide insights into the accurate measurement of the output of the variable impedance energy device of a TENG, which is crucial for the optimization of the device to maximize its power utilization in real-world applications.

## Experimental

### Fabrication of the CS-TENG

In the experimental procedure, a contact-separate TENG (CS-TENG) was fabricated with a contact area defined by adhering a 2 × 2 cm^2^ polymethyl methacrylate (PMMA) plate to a 4 × 4 cm^2^ PMMA substrate. The 50 μm-thick aluminum tape was used as the electrode on both sides of the two facing contact areas, while the 50 μm-thick layer of polyimide tape was adhered on the side of the aluminum electrode to serve as the dielectric layer. The charge density of the TENG was enhanced using the prior charge injection method, which utilizes an electric field formed by a high-voltage source [21]. Following exposure to an electric field for a duration, the surface voltage of the dielectric remains constant even after the electric field is removed. The output voltage was maintained at 93% after 500 s of exposure to air (Fig. S1). The experiment was conducted at 22 °C and 46% RH.

### Output measurement of the TENG

**Fig. 1:**
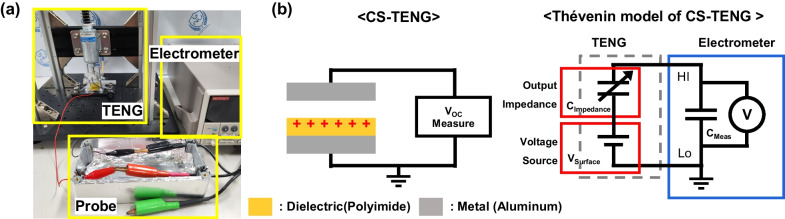
**a** Photograph of the TENG and the *V*_*OC*_ measurement system. **b** Schematics of the CS-TENG and equivalent model of the *V*_*OC*_ measurement system

The contact-separation process of the CS-TENG was repeated using a solenoid (Shindengen, M080117SS). A force sensor (Marveldex, RA18) was positioned beneath the CS-TENG to detect consistent force application. There are two ways to measure open-circuit voltage: (1) a voltmeter (contact method) and (2) an electrostatic field meter (noncontact method). The *V*_*OC*_ is measured as the voltage without a connected load between the device and the voltmeter. An ideal voltmeter should remain open-circuited; it should not draw any current. To minimize the current flowing through the measuring device, an electrometer with a high impedance is necessary. The electrical output characteristics of the TENG with initial charge balancing were measured using an electrometer (Keithley, 6514). The value of 370 pF was measured in the electrometer and probe measurement system (*C*_*Meas*_) using a capacitance meter (FLUKE, 289). An electrostatic field meter has the advantages of virtually zero input capacitance and infinite impedance. We used an electrostatic field meter (SIMCO, FMX-004) for noncontact voltage measurements. For the two methods, the analog output was connected to an oscilloscope (Keysight, DSO-2014A) to obtain the waveform. Figure 1a shows the experimental setup. Figure 1b illustrates the structure of the CS-TENG and a schematic of the *V*_*OC*_ measurement.

### Equivalent circuit simulation

The Multisim program (National Instruments Inc.) was used as the Simulation Program with Integrated Circuit Emphasis (SPICE) to model the behavior of the TENG. Figure 1b shows the lumped-parameter equivalent model of the TENG and measurement system, which consists of three components: constant surface voltage (*V*_*Surface*_), intrinsic output series impedance (*C*_*Impedance*_), and measurement system impedance (*C*_*Meas*_). Many studies have used this model to understand the output of TENGs [22–24]. The *V*_*Surface*_ of the TENG formed by the dielectric layer determines the output of the TENG according to the change in the *C*_*Impedance*_ of the TENG during the contact-separation process. The electrical output characteristics were measured by connecting the HI and LO probes of an electrometer. This process connects the input capacitance of the electrometer (*C*_*Meas*_) to the TENG. The SPICE was used with an equivalent model to simulate the output of the TENG. The values used in the simulation were set as follows. The capacitance of the TENG in the contact separation process was obtained via calculations. The contact capacitance is 233.7 pF, and the isolation capacitance is 0.554 pF. The value of *V*_*Surface*_ was set to 134 V, which is the saturation voltage from the experiment. We confirmed that the saturation voltage determines the *V*_*Surface*_ of the lumped parameter model of the TENG when a half-wave rectifier is used [25]. The ideal device models used in the SPICE simulation were as follows: the diode has no reverse breakdown voltage, the forward threshold voltage is 1 V, and there was no leakage and infinite conductance in the wire. The parameters used in the SPICE model (Table S1) and the measured *V*_*Surface*_ can be found in Fig. S2.

## Results and discussion

### Dual polarity open circuit voltage in TENG

In the idealized state of an open circuit, the output voltage produced by the *V*_*Surface*_ and *C*_*Impedance*_ should be the same as the *V*_*Surface*_. However, under actual measuring conditions, the *C*_*Impedance*_ of the TENG and *C*_*Meas*_ of the measurement system are connected in series, dividing the *V*_*Surface*_ to a certain voltage that is less than the *V*_*Surface*_. When measuring voltage with an electrometer, an initial voltage reference of 0 V must be set initially, which is called a zero check. In this process, an internal shunt resistor of 10 MΩ of our electrometer (Keithley 6514) is connected between the measuring probes to equilibrate (zero-check), which is a charge balancing between the TENG and the measurement system; the shunt resistance value is referenced from the zero-check section of the manual [26]. This phenomenon can be replicated with a shunting wire instead of an internal wire of 10 MΩ. The amount of charge stored in the *C*_*Meas*_ will balance the source voltage with the voltage reference. There are two types of balancing processes: the separate balancing process (SBP), in which the capacitors of the measurement system are charged and balanced while the TENG is separated, and the contact balancing process (CBP), in which the capacitors of the measurement system are charged and balanced while the TENG is in contact. It should be noted that these SBP and CBP states have two distinctive *C*_*Impedance*_ conditions.

**Fig. 2:**
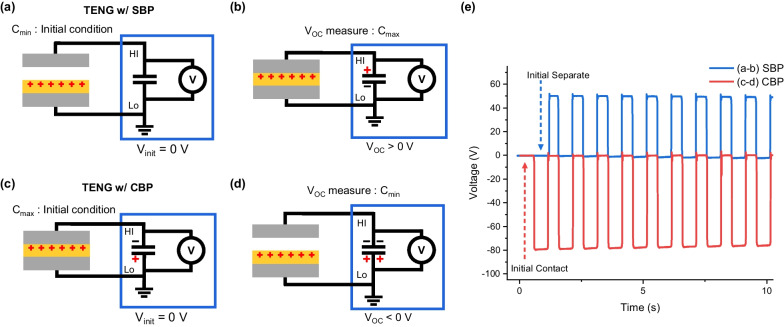
Illustration of the initial impedance state of the TENG for setting the reference voltage and charge depiction during measurement. **a** Setting the reference voltage at *C*_*min*_ (SBP) and **b** measuring at *C*_*max*_. **c** Setting the reference voltage at *C*_*max*_ (CBP) and **d** measuring at *C*_*min*_. **e** Voltage measurement results depending on the initial conditions

In the initial condition with SBP, the output capacitance is minimum (*C*_*min*_, Fig. 2a). We measure the *V*_*OC*_ when the output capacitance is maximum (*C*_*max*_, Fig. 2b). In the initial condition with the CBP, the output capacitance is maximum (*C*_*max*_, Fig. 2c). We measure *V*_*OC*_ when the output capacitance is minimum (*C*_*min*_, Fig. 2d). Figure 2e shows the measured voltage starting from the reference voltage of zero volt depending on the initial CBP and SBP conditions. When the dielectric layer, which is the friction layer of the TENG, bears a positive surface voltage, the output voltage of the TENG would be larger than the voltage at the separate state (*C*_*min*_). It has a maximum voltage at the contact state (*C*_*max*_) regardless of the initial balancing process. Thus, the polarity of the output voltage is positive after SBP and negative after CBP, and the magnitude of the *V*_*OC*_ is 82 V with CBP, which is approximately 1.6 times larger than that of 52 V with SBP. This resulted in two measured *V*_*OC*_s with different magnitudes and different polarities depending only on the initial balancing process, i.e., setting the initial reference voltage.

**Fig. 3:**
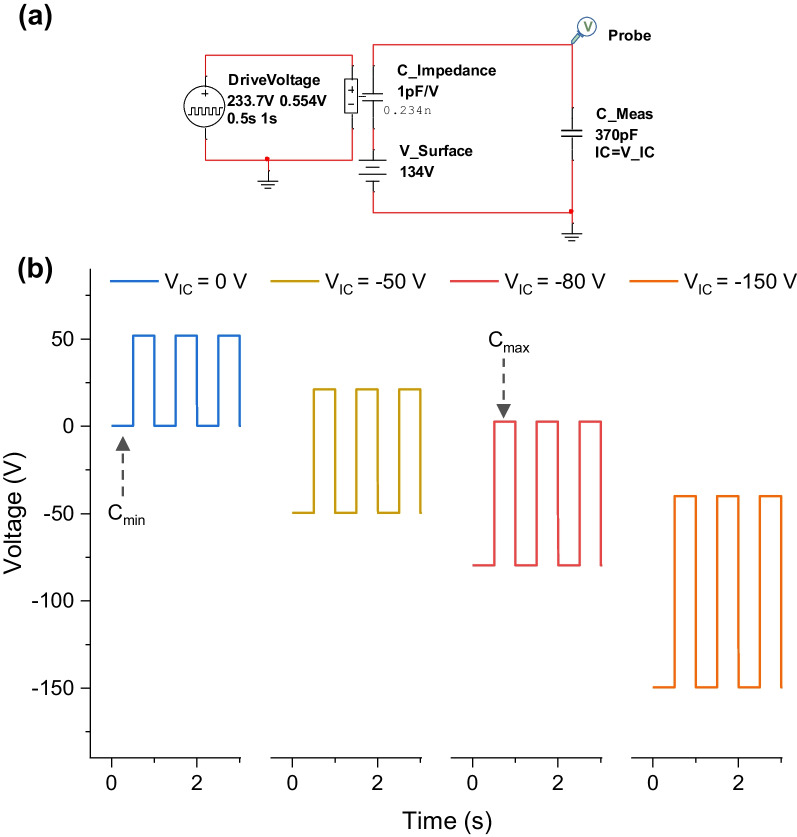
**a** SPICE model according to the initial voltage condition (*V*_*IC*_) of *C*_*Meas*_. **b** Output voltage for each initial voltage condition. The first lower state represents the initial voltage at the separate state (*C*_*min*_). The ascending peak indicates the voltage at the contact state (*C*_*max*_)

A circuit simulation was performed based on the equivalent circuit model parameters using the SPICE to understand the difference in *V*_*OC*_ depending on the initial condition of charge balancing. In Fig. 3a, *C*_*Meas*_ has an initial voltage condition (IC) to model the initial charge stored during the initial balancing process. When the *C*_*Meas*_ has an initial voltage (*V*_*IC*_) of zero, there are no initially stored charges at *C*_*Meas*,_ which means that there are almost zero bound charges at the electrodes of the TENG. Thus, *V*_*Suface*_ will be divided by the *C*_*Impedance*_ and *C*_*Meas*_ rendering output voltages. This is the case for the SBP with the minimum capacitance of the TENG (i.e., a voltage of zero under separate condition). If initially stored charges exist in *C*_*Meas*_, the output voltage can be interpreted as a superposition of two sources: the *V*_*Surface*_ and initial stored charges at *C*_*Meas*_. The stored charges in *C*_*Meas*_ will be divided by the *C*_*Impedance*_ and *C*_*Meas*_. Thus, the measured voltage will be modified (shifted) from the SBP case depending on the amount of stored charges in *C*_*Meas*_ (i.e., *V*_*IC*_) (Fig. 3b). For an initial voltage (*V*_*IC*_) of 0 V, the output voltage is 52 V_pk-pk_; for − 50 V, it is 71 V_pk-pk_; for − 80 V, it is 83 V_pk-pk_; and for − 150 V, it is 110 V_pk-pk_. Among them, there is a unique state where the voltage generated from the *V*_*Surface*_ is complemented by the voltage generated from charge redistribution originating from the *V*_*IC*_. In this state, the output will be zero. Thus, the voltage swing of the measurement result will be from the *V*_*IC*_ to zero. Zero output will occur at the maximum charge redistribution state. If we start from the contact state, the maximum charge redistribution will occur in the separate state and vice versa. Because we want to simulate the CBP condition (i.e., a voltage of zero at the contact condition), we must choose the voltage minimum (i.e., *V*_*IC*_) under separate condition. Thus, we know that the initial separate state with zero *V*_*IC*_ causes SBP, and the initial separate state with a certain negative *V*_*IC*_ can cause CBP. In the simulation, this condition corresponds to a voltage condition of *V*_*IC*_ = −82 V. With the aid of a negative *V*_*IC*,_ we successfully simulated SBP and CBP by the motion of separate or contact in a unified form, which is consistent with the experimental results (*V*_*OC*_ at SBP: 52 V, *V*_*OC*_ at CBP: − 82 V).

### Model of repeated charge balancing with diode

**Fig. 4:**
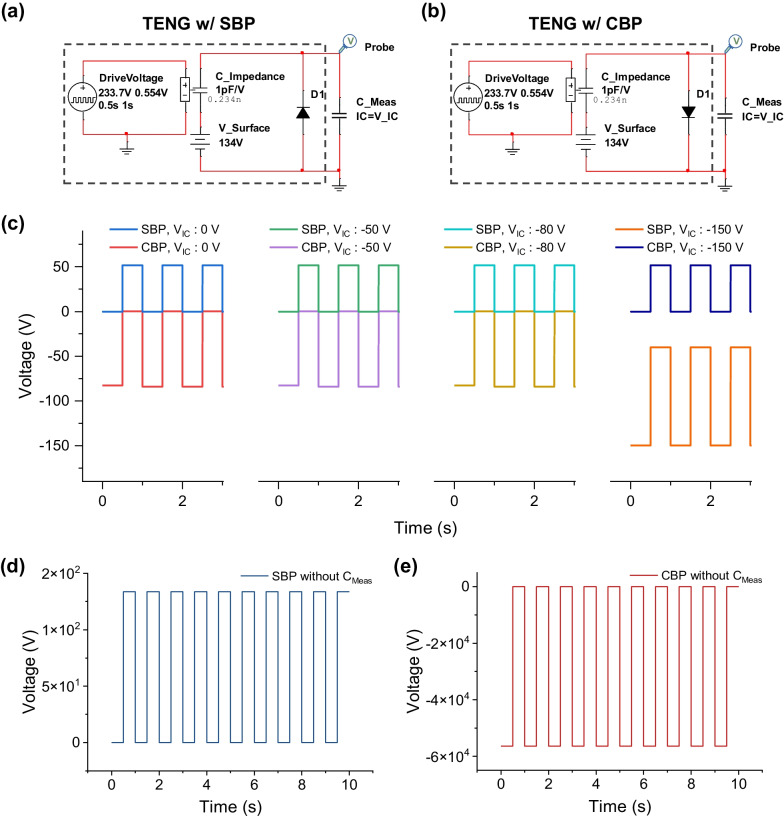
SPICE model with initial reference voltage conditions using a diode connection. **a** TENG with SBP condition. **b** TENG with CBP condition. (c) Output voltage depending on the initial reference voltage conditions with different initial charged *C*_*Meas*_ voltages (*V*_*IC*_). Output of the TENG in the absence of a measurement system (*C*_*Meas*_ = 0 pF) under **d** SBP and **e** CBP conditions

It is difficult to set an initial condition in which the charges in *C*_*Impedance*_ and *C*_*Meas*_ are balanced to produce a reference voltage of 0 V in the CBP condition in the SPICE. When a diode, which is an active element, is connected to both ends of the TENG, the potential difference between the two nodes becomes the forward voltage of the diode (*V*_*F*_ = 1 V). It can be treated as a zero volt (compared to the high voltage output of the TENG). Due to the diode being turned on in the forward voltage direction, we set the reference voltage in this turn-on period, and the reverse voltage can be maintained referenced from the diode turn-on condition. The TENG with a positive output voltage referenced from zero volt (*C*_*min*_) can be simulated with Fig. 4a. Similarly, a TENG with a negative output voltage referenced from zero volt (*C*_*max*_) can be simulated with Fig. 4b. With the same SPICE parameters used in Fig. 3, we can obtain the same results for SBP and CBP, even under different *V*_*IC*_ conditions. If we choose the initial condition above the *V*_*IC*_ of − 82 V, we can obtain consistent results, as shown in Fig. 4c. When the *V*_*IC*_ is lower than − 82 V, the voltage generated from the maximum charge redistribution will be larger than that generated from the *V*_*Surface*_. Therefore, the overall voltage output will be negative in this case (e.g., *V*_*IC*_ = −150 V), and the balancing process cannot be simulated. For this reason, we can safely and conveniently choose zero *V*_*IC*_ conditions in all CBP and SBP cases. In the simulation, diodes were connected to replicate the initial balancing effect. Regardless of the initial voltage conditions of *C*_*Meas*_, the occurrence of SBP and CBP is solely based on the direction of the diodes, which can be interpreted as repeated charge balancing in each cycle. To verify the repeated charge balancing, we removed the initial charge balancing effect by setting *C*_*Meas*_ = 0 pF, i.e., no measurement system. Even at this time, two different *V*_*OC*_s were observed, indicating that any form of charge balancing effect still existed, as confirmed in Fig. 4d, e. When the diode turn on in each cycle, the two electrodes became the same potential, which is a charge balancing effect as previously described in Sect. 3.1. Additionally, the charge dividing effect caused by *C*_*Meas*_ is eliminated, resulting in an increased voltage.

### Open-circuit voltages of the TENG with a capacitive load

**Fig. 5:**
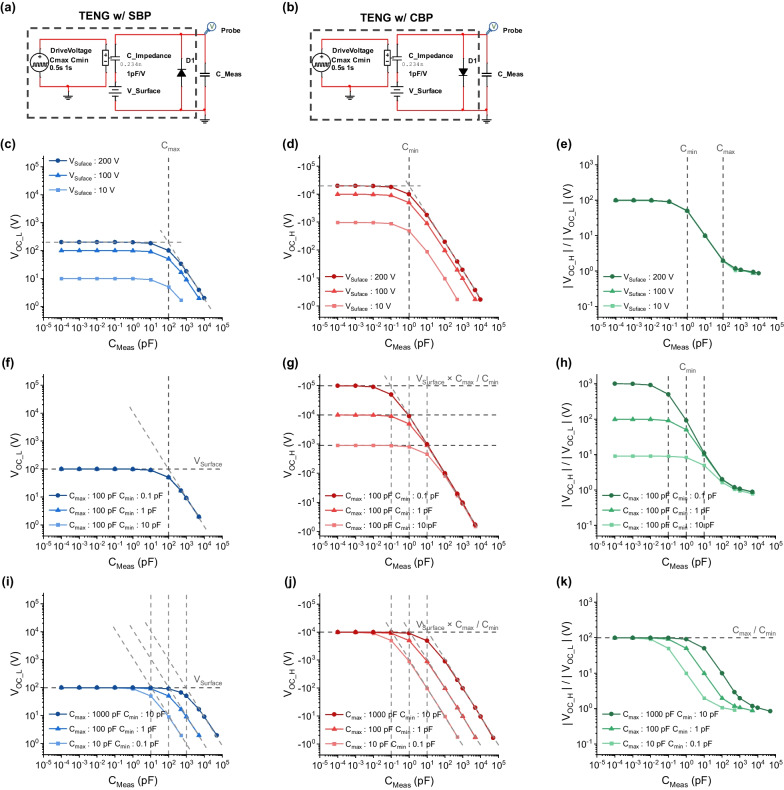
*V*_*OC*_ with different *C*_*Meas*_ in the repeated charge balancing. TENG with **a** SBP and **b** CBP conditions connected to the measurement system. The *V*_*OC*_s of the TENG with changes in surface voltage (*V*_*Surface*_). **c** Lower *V*_*OC*_ (*V*_*OC_L*_), **d** higher *V*_*OC*_ (*V*_*OC_H*_), and **e** the ratio of *V*_*OC_H*_ to *V*_*OC_L*_ at fixed values of *C*_*min*_ = 1 pF and *C*_*max*_ = 100 pF when *V*_*Surface*_ is varied. At a constant *V*_*Surface*_ of 100 V with a fixed *C*_*max*_, the changes in (f) *V*_*OC_L*_, **g** *V*_*OC_H*_, and **h** the ratio of *V*_*OC_H*_ to *V*_*OC_L*_ are shown as a function of *C*_*Meas*._ The effect of varying the magnitude of *C*_*max*_ and *C*_*min*_ while maintaining the ratio of *C*_*max*_ to *C*_*min*_ fixed to 100 with a *V*_*Surface*_ of 100 V, **i** *V*_*OC_L*_, **j** *V*_*OC_H*_, and **k** the ratio of *V*_*OC_H*_ to *V*_*OC_L*_ as a function of *C*_*Meas*_

Since it was confirmed that *C*_*Meas*_ reduces *V*_*OC*_ due to the charge dividing effect, *V*_*OC*_ was analyzed by varying *C*_*Meas*_ to systematically investigate this phenomenon. Depending on the type of balancing process, a TENG with a specific balancing condition can be represented as shown in Fig. 5a, b. The polarity of the voltage output can be chosen according to the direction of the diode. To simulate the input impedance of the voltage measurement system, we add *C*_*Meas*_ to the ideal voltage probe of the SPICE. Figure 5c–e shows the results of varying *V*_*Surface*_ while keeping *C*_*max*_ and *C*_*min*_ fixed. The positive voltage (SBP), *V*_*OC_L*,_ follows the *V*_*Surface*_ when *C*_*Meas*_ is small enough. As the *C*_*Meas*_ increases, impedance matching occurs at the point where *C*_*Meas*_ equals the series output impedance *C*_*max*_, reducing the voltage to 1/2 of the original *V*_*Surface*_. If *C*_*Meas*_ becomes large enough, *V*_*OC_L*_ rolls off (Fig. 5c). In the case of the CBP, we obtain a negative *V*_*OC_H*_. The absolute value of *V*_*OC_H*_ is larger than that of *V*_*Surface*_. The increased output voltage is maintained if the *C*_*Meas*_ is small enough (Fig. 5d). The dependency on *C*_*Meas*_ is equal to that of *V*_*OC_L*_. Impedance matching occurs when *C*_*Meas*_ is equal to *C*_*min*_, which is the output impedance of the TENG under measurement conditions (Fig. 2d). The ratio of *V*_*OC_H*_ to *V*_*OC_L*_ is constant regardless of the *V*_*Surface*_, which is the value of *C*_*max*_*/C*_*min*_ when *C*_*Meas*_ is small enough (Fig. 5e), but this ratio steadily decreases as *C*_*Meas*_ becomes larger than *C*_*min*_. This ratio can be termed the amplification ratio.

Only *C*_*min*_ was varied to verify this amplification ratio, while *V*_*Suface*_ and *C*_*max*_ were fixed, as shown in Fig. 5f–h. The output voltage is constant with the value of the *V*_*Surface*,_ and the role of the point is also fixed at the corresponding series impedance of *C*_*max*_ (Fig. 5f). We can see that *V*_*OC_L*_ is independent of *C*_*min*_. On the other hand, by varying *C*_*min*_, it was observed that three different values of *V*_*OC_H*_ were obtained by three different *C*_*min*_ at small enough *C*_*Meas*,_ and the impedance matching point followed each *C*_*min*_ value (Fig. 5g). Each *V*_*OC_H*_ can be obtained by *V*_*Surface*_ × *C*_*max*_/*C*_*min*_. Thus, the ratio of *V*_*OC_H*_ to *V*_*OC_L*_ follows the *C*_*max*_/*C*_*min*_ ratio precisely as before (Fig. 5h).

As shown in Fig. 5i–k, the *C*_*max*_ and *C*_*min*_ varied simultaneously at the same rate but with a fixed ratio of *C*_*max*_/*C*_*min*_ = 100. We can see that *V*_*OC_L*_ and *V*_*OC_H*_ are fixed at constant values at sufficiently small *C*_*Meas*_ and impedance matching with each *C*_*min*_ and *C*_*max*_ (Fig. 5i, j). The *V*_*OC_H*_/*V*_*OC_L*_ ratio follows the *C*_*Impedance*_ ratio of *C*_*max*_/*C*_*min*_ when the *C*_*Meas*_ is less than *C*_*min*_ (Fig. 5k).

When the influence of *C*_*Meas*_ is very small, the two *V*_*OC_L*_ and *V*_*OC_H*_ values converge to constant values, indicating that these are intrinsic characteristics of the TENG. Under SBP conditions, the amount of charge accumulated during the charge balancing process facilitated by the diode is determined by *C*_*min*_ and *V*_*Surface*_.

1 $$\begin{array}{*{20}c} {Q_{SBP} = C_{min} \times V_{Surface} } \\ \end{array}$$

When the charged TENG with the *Q*_*SBP*_ transit to a contact state, the output voltage is the sum of the voltage generated by the *Q*_*SBP*_ and the *V*_*Surface*_.

2 $$\begin{array}{*{20}c} {V_{OC\_L} = \frac{{Q_{SBP} }}{{C_{max} }} + V_{Surface} = \frac{{C_{min} \times V_{Surface} }}{{C_{max} }} + V_{Surface} } \\ \end{array}$$

When *C*_*min*_/*C*_*max*_ is negligible in Eq. (2), *V*_*OC_L*_ can be expressed as follows:

3 $$\begin{array}{*{20}c} {V_{OC\_L} = \left( {\frac{{C_{min} }}{{C_{max} }} + 1} \right) \times V_{Surface} \cong V_{Surface}\qquad\left( {C_{min} /C_{max} \ll 1} \right) } \\ \end{array}$$

Under CBP conditions, the amount of charge accumulated during the charge balancing process facilitated by the diode is as follows:

4 $$\begin{array}{*{20}c} {Q_{CBP} = C_{max} \times V_{Surface} } \\ \end{array}$$

The output generated by *Q*_*CBP*_ and *V*_*Surface*_ is as follows:

5 $$\begin{array}{*{20}c} {V_{OC\_H} = \frac{{Q_{CBP} }}{{C_{min} }} + V_{Surface} = \left( {\frac{{C_{max} }}{{C_{min} }} + 1} \right) \times V_{Surface} } \\ \end{array}$$

When *C*_*max*_/*C*_*min*_ is very large, the output voltage can be expressed as follows:

6 $$\begin{array}{*{20}c} {V_{OC\_H} \cong \frac{{C_{max} }}{{C_{min} }} \times V_{Surface} \qquad\left( {1 \ll C_{max} /C_{min} } \right)} \\ \end{array}$$

When the balancing process occurs under CBP conditions facilitated by the diode, a charge corresponding to the *V*_*Surface*_ is stored in *C*_*max*_. Subsequently, as the impedance changes to *C*_*min*_, an additional voltage is generated by the previously stored charge, resulting in a higher output voltage than that of the *V*_*Surface*_.

### Experimental verification of diode balancing

**Fig. 6:**
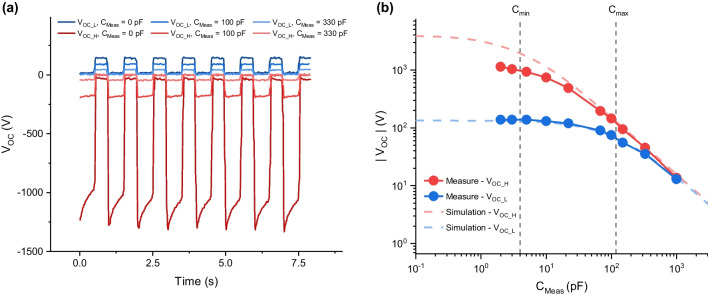
**a** *V*_*OC_L*_ and *V*_*OC_H*_ were measured using an electrostatic field meter with an additional capacitor as *C*_*Meas*_. **b** Measurement and simulation results. The SPICE parameters were modified by including the diode effect

We can see that *V*_*OC_L*_ is determined by *V*_*Surface,*_ and its series impedance is *C*_*max*_, while *V*_*OC_H*_ has a value of *C*_*max*_/*C*_*min*_ × *V*_*Surface*_, and its series impedance is *C*_*min*_. The output properties of TENG are determined by its two impedances, and to achieve a high output voltage, a balancing diode must be connected. To fulfill these two conditions, two characteristic impedances, *C*_*min*_ and *C*_*max,*_ must include the balancing diode effect. To verify this, the *C*_*min*_ and *C*_*max*_ of the TENG with a diode were measured (Fig. S3). Using these parameters (*V*_*Surface*_ = 138 V, *C*_*min*_ = 4 pF, and *C*_*max*_ = 118 pF), the changes in *V*_*OC*_ with varying *C*_*Meas*_ were measured and compared with circuit simulations. We minimized the measurement capacitance by using a noncontact voltage measurement device such as an electrostatic fieldmeter, and we added an additional capacitor to simulate the *C*_*Meas*_. In the circuits used for the experiment shown in Fig. 5a, b, the voltage of the TENG was measured (Fig. 6a). Figure 6b shows the measurement and simulation results. The experimental outputs of *V*_*OC_L*_ and *V*_*OC_H*_ are consistent with the simulation results. However, *V*_*OC_H*_ slightly decreases compared with the simulation results. This can be originated from the decreased voltage, as shown by the sawtooth shape at *C*_*Meas*_ = 0 pF (Fig. 6a). In the SPICE simulation, the parameter of the RSHUNT represents the leakage of the DC path between the circuit and the ground. The output (Fig. S4) replicates a sawtooth shape with this finite leakage parameter. Due to leakage, it can be expected that the observed voltage would be lower than the ideal simulation results without leakage. When we conducted initial charge balancing with *C*_*Meas,*_ the outputs of SBP and CBP converged to a value between that of CBP and SBP over time. In contrast, when we apply repeated charge balancing with a diode, the output voltage decreases in each cycle but remains constant across the repeating cycles. The maintenance of the reference voltage level came from the recovery charge originated from the ground.

### TENG power curve for the resistive load

**Fig. 7:**
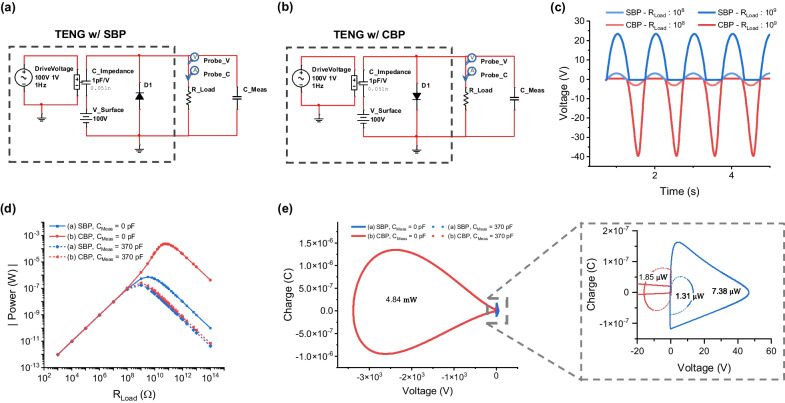
SPICE models of a TENG under resistive load conditions with **a** SBP and **b** CBP reference voltage settings. The *C*_*Impedance*_ varies sinusoidally at 1 Hz between *C*_*max*_ = 100 pF and *C*_*min*_ = 1 pF. **c** Output voltage waveform with two different resistive loads. **d** The power curve as a function of resistive load. **e** Q–V plot at the maximum power output point of **d**, where the numerals represent the area of the curve, indicating the output power

In order to verify that *V*_*OC_H*_ has a physical meaning, we calculated the power curve for the resistive load. To observe the power curve, we drive the TENG in a sinusoidal shape at 1 Hz with a corresponding sinusoidal change in the series output impedance with a maximum value of 100 pF and a minimum value of 1 pF. The circuits in Fig. 7a, b have a resistive load without or with (dashed line) *C*_*Meas*_ of the measurement system. For the two different resistive loads without *C*_*Meas*_, a half-wave sinusoidal voltage appears across the resistive load depending on the SBP and CBP conditions (Fig. 7c). The output voltage and current were used to determine the output power of the TENG (Fig. 7d, e). From the behavior of the same TENG, the maximum output power is observed at different points depending on the orientation of the diode. The difference between these maximum power outputs is approximately 320 times when *C*_*Meas*_ is zero (Fig. 7d). When measured using *C*_*Meas*_ of 370 pF with a resistive load, the output power lines almost coincide, as shown in the two lower output lines in Fig. 7d. With an ideal voltage measurement system with a very small *C*_*Meas*_ = 0 pF, all the internal power of the TENG is delivered to the resistive load. However, in the real world, when the voltage measurement system has infinite resistance but finite capacitance, the impedance of the instrument system affects the circuit, as shown in Fig. 5. The output characteristics under the CBP condition severely deteriorate. The charge transfer during the operation of the TENG at the point of maximum power output was obtained, as shown in the Q–V plot in Fig. 7e. The Q–V plot also shows a large output of CBP (4.84 mW) and SBP (7.38 μW) in the ideal situation of *C*_*Meas*_ = 0 pF. In the case of *C*_*Meas*_ = 370 pF, we can see that both the CBP (1.85 μW) and SBP (1.31 μW) in the zoomed Q–V plot are heavily deteriorated and almost similar sized circular shape output characteristics as in the case of the lower power curves of Fig. 7d. If we do not deteriorate the output of the TENG with significantly smaller *C*_*Meas*_ (i.e., parasitic capacitive load in a without measurement system), we can effectively use the maximum power output by selecting an appropriate initial/repeated balancing process.

## Conclusions

In our experiments, two distinctive *V*_*OC*_s were observed, which were dependent on the initial voltage setting method. This behavior was replicated in circuit simulations, where the output voltage depended on the initial voltage setting method. Furthermore, we successfully achieved two independent *V*_*OC*_s in the simulation and measurement by employing diode connections, irrespective of the initial voltage settings. The diode connection can be interpreted as repeated charge balancing.

When the measurement system capacitance was lower than the minimum series capacitance, the *V*_*OC_L*_ was equivalent to the *V*_*Surface*_. Meanwhile, the *V*_*OC_H*_ was amplified by the product of the *V*_*Surface*_ and the ratio of the TENG's characteristic impedance (*C*_*max*_/*C*_*min*_). However, as the measurement system impedance increased, the difference between the two *V*_*OC*_s decreased, and their absolute magnitudes also fell below the *V*_*Surface*_.

The system exhibited two distinct power characteristics when a resistive load was connected. To maximize the output power, it is essential to utilize the amplified *V*_*OC*_. This necessitates precise reference voltage settings and minimizing the capacitive load of the measurement system or parasitic component to less than the *C*_*min*_ of the TENG.

This study highlights the critical influence of the capacitance of the system on the performance of the TENG. This finding underscores the necessity of proper reference voltage settings (initial/repeated charge balancing) to fully leverage the potential of TENGs for various applications.

### Supplementary Information

Additional file1 (DOCX 162 kb)

## Data Availability

The data are provided within the manuscript or supplementary information files.

## Abbreviations

TENG: Triboelectric nanogenerator
*V*_*OC*_: Open-circuit voltage
*I*_*SC*_: Short-circuit current
CS-TENG: Contact-separate mode TENG
PMMA: Polymethyl methacrylate
SPICE: Simulation Program with Integrated Circuit Emphasis
*V*_*Surface*_: Surface voltage
*C*_*Impedance*_: Intrinsic output series impedance
*C*_*Meas*_: Measurement system impedance
SBP: Separate balancing process
CBP: Contact balancing process
*C*_*min*_: Minimum capacitance of TENG
*C*_*max*_: Maximum capacitance of TENG
IC: Initial voltage condition
*V*_*IC*_: Initial voltage
*V*_*F*_: Forward voltage of the diode
