# Supplementary material for: Dual polarity open circuit voltage in triboelectric nanogenerators originated from two states series impedance
Source: Discov Nano. 2024 Jul 6;19(1):111. doi: 10.1186/s11671-024-04056-y (PMC11227483; doi:10.1186/s11671-024-04056-y)
Supplement: Supplementary file 1 — Additional file1 (DOCX 162 kb) [file 11671_2024_4056_MOESM1_ESM.docx]

**Supplementary Information**

**Dual Polarity Open Circuit Voltage in Triboelectric Nanogenerators Originated from Two States Series Impedance**

Jiwon Jeong, Jiyoung Ko, Jongjin Lee^*^

Department of Physics and Research Institute of Natural Science, Gyeongsang National University, Jinju 52828, South Korea

* Author to whom correspondence should be addressed (J. Lee)

Tel.: +82-55-772-1401

E-mail address: bandy1@gnu.ac.kr

Fig. S1. Time-dependent output voltage under initial CBP and SBP conditions.

**Table S1**. Parameters of the TENGs utilized in the SPICE model.

| **Parameter** | **Value** |
| --- | --- |
| Permittivity | $\epsilon_{0}=8.854\times{10}^{12} F/m$ |
| Dielectric 1 (Polyimide) | $\epsilon_{1}=3.3, d_{1}=50 \mu m$ |
| Dielectric 2 (Air) | $\epsilon_{2}=1.00059, d_{2}=6.38 \mathrm{mm}$ |
| Contact Area | $S=4\times{10}^{-4}m^{2}$ |
| Surface Voltage | 134 V |
| Contact, Separate transient time | 10 ms |
| C_min_ | $0.554 \mathrm{pF}$ |
| C_max_ | $233.7 \mathrm{pF}$ |

The contact and separation capacitance (C_max_ and C_min_) of the TENG can be calculated from the following equation:

$$\begin{aligned} \boldsymbol{C=}\frac{\boldsymbol{\epsilon}_{\boldsymbol{0}}\boldsymbol{\times S}}{\frac{\boldsymbol{d}_{\boldsymbol{1}}}{\boldsymbol{\epsilon}_{\boldsymbol{1}}}\boldsymbol{+}\frac{\boldsymbol{d}_{\boldsymbol{2}}}{\boldsymbol{\epsilon}_{\boldsymbol{2}}}}\boldsymbol{\#}\left( \mathbf{S}\mathbf{1} \right) \end{aligned}$$

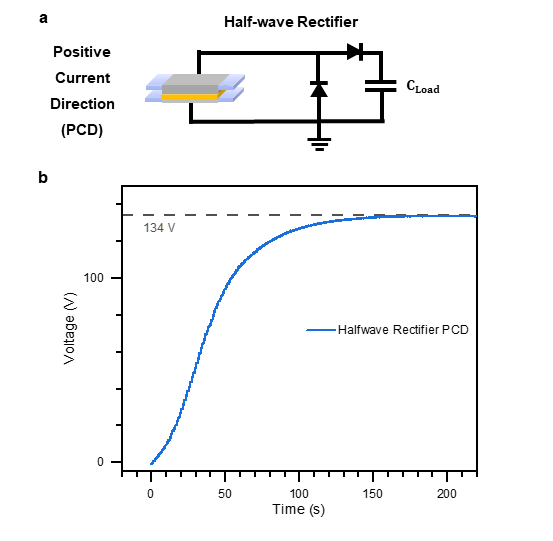


Fig. S2. (a) Experimental TENG circuit diagrams with a capacitive load through a half-wave rectifier. (b) The voltage appeared across the load capacitor of C_Load_ = 47 nF in the circuit half-wave rectifier.

When the positive surface voltage of the TENG's dielectric layer, we term the positive direction as the current flows from the positive voltage of V_Surface_ voltage to C_Load_. Connected to a half-wave rectifier with a positive current direction (PCD), the saturation voltage of the capacitor means the V_Surface_ of the TENG [1].

[1] J. Jeong, J. Ko, J. Kim, and J. Lee, Asymmetric voltage amplification using a capacitive load energy management circuit in a triboelectric nanogenerator, Discov Nano **19** (2024) 52. https://doi.org/10.1186/s11671-024-03997-8


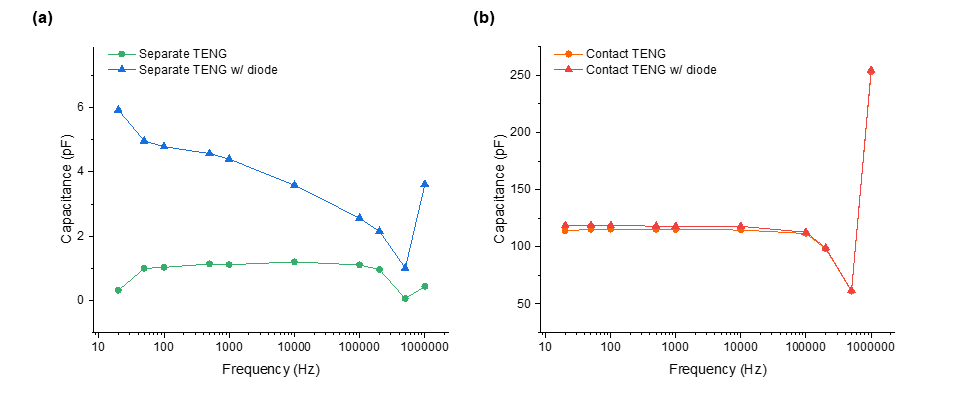


Fig. S3. Capacitance measurement of the TENG with the balancing diode (FV5M-08) at different frequencies in (a) the separated state and (b) the contact state. An LCR meter (4284A, Hewlett Packard) was used with a driving amplitude of 1 V.


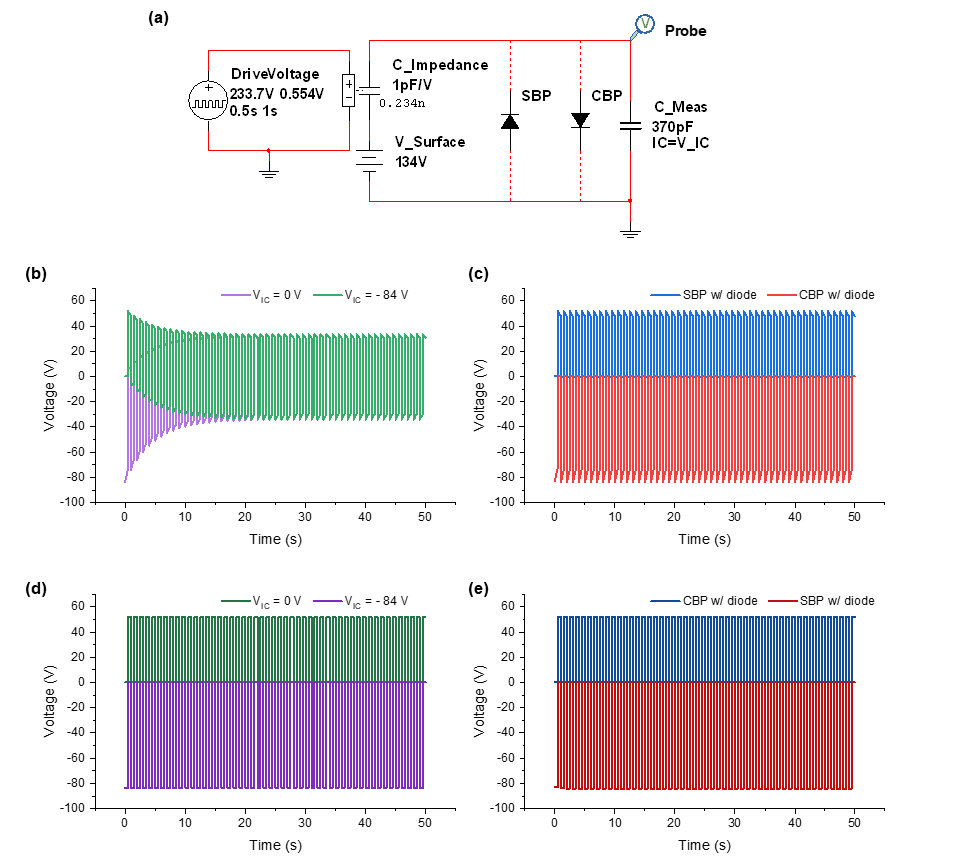


Fig. S4. Simulation to investigate the effect of leakage on TENG output. The RSHUNT value in SPICE was adjusted to model the leakage between the circuit and ground [2]. (a) Circuit and parameters used in the simulation. The dotted line with the diode is connected in the appropriate direction under each SBP or CBP condition. When RSHUNT = 10^10^ Ω, (b) the output voltage of the TENG with initial charge balancing of CMeas, and (c) the output voltage of the TENG with repeated charge balancing by using the diode. When RSHUNT = ∞ Ω, (d) the output voltage of the TENG with initial charge balancing of CMeas, and (e) the output voltage of the TENG with repeated charge balancing by using the diode.

[2] N. Instruments and E. W. Group, NI Multisim User Manual 2009), p.^pp. 1-814
